# SUMOylation of TBL1 and TBLR1 promotes androgen-independent prostate cancer cell growth

**DOI:** 10.18632/oncotarget.9002

**Published:** 2016-04-26

**Authors:** Soo-Yeon Park, Younghwa Na, Mee-Hee Lee, Jae-Sung Seo, Yoo-Hyun Lee, Kyung-Chul Choi, Hyo-Kyoung Choi, Ho-Geun Yoon

**Affiliations:** ^1^ Department of Biochemistry and Molecular Biology, Brain Korea 21 PLUS Project for Medical Sciences, Yonsei University College of Medicine, Seodaemun-gu, Seoul, South Korea; ^2^ College of Pharmacy, CHA University, Gyeonggi-do, Pocheon, South Korea; ^3^ Department of Food Science and Nutrition, The University of Suwon, Kyunggi-do, South Korea; ^4^ Department of Biomedical Sciences, University of Ulsan College of Medicine, Poongnap-dong, Songpa-gu, Seoul, South Korea; ^5^ Division of Nutrition and Metabolism Research Group, Korea Food Research Institute, Gyeonggi-do, South Korea

**Keywords:** SUMOylation, TBL1, TBLR1, NF-κB, inflammation

## Abstract

Chronic inflammation is strongly associated with prostate cancer pathogenesis. Transducin β-like protein (TBL1) and Transducin β-like 1X-linked receptor 1 (TBLR1) have been identified recently as a coactivator for NF-κB-mediated transcription; however, the underlying mechanism by which TBL1 and TBLR1 activate NF-κB function during inflammation remains unknown. Here, we demonstrate that cytokine production is significantly elevated in androgen-independent PC-3 prostate cancer cells compared with androgen-dependent LNCaP prostate cancer cells. Elevated cytokine production positively correlates with the TBL1 and TBLR1 SUMOylation level in PC-3 cells. We show that both TBL1 and TBLR1 are SUMOylated in response to TNF-α treatment, and this increases formation of the TBL1-TBLR1-NF-κB complex, which leads to NF-κB-mediated transcriptional activation of cytokine gene expression. Conversely, SENP1-mediated deSUMOylation of TBL1 and TBLR1 inhibits NF-κB-target gene expression by dissociating TBL1 and TBLR1 from the nuclear hormone receptor corepressor (NCoR) complex. TBL1 knockdown substantially suppresses inflammatory signaling and PC-3 cell proliferation. Collectively, these results suggest that targeted SUMOylation of TBL1 and TBLR1 may be a useful strategy for therapeutic treatment of androgen-independent prostate cancer.

## INTRODUCTION

Chronic inflammation leads to a wide variety of diseases, such as rheumatoid arthritis, other autoimmune disorders, cardiovascular disease, gastrointestinal disorders, and several cancers [1, 2]. Prostate cancer (PCa) is the most commonly diagnosed malignancy in men [3, 4]. Chronic inflammation is one of the most important factors in prostate cancer etiology and carcinogenesis, and plasma IL-6 level has prognostic significance in patients with hormone-refractory prostate cancer [5, 6]. Recently, a five-year follow-up study for men who had abnormal serum prostate-specific antigen (PSA) levels and/or digital rectal examinations (DRE) reported that chronic inflammation is a significant risk factor in the development of prostate cancer [7]. Therefore, blocking inflammatory signaling may be a useful strategy for treating malignant prostate cancer.

NF-κB is a pleiotropic transcription factor that controls the expression of numerous genes encoding cytokines, angiogenesis modifiers, cell adhesion molecules, and anti-apoptotic factors [8]. The mammalian NF-κB family has five members: RelA (p65); NF-κB1 (p50, p105); NF-κB2 (p52, p100); c-Rel; and RelB. The most abundant activated form of NF-κB is a heterodimer that contains p65 and p50 subunits. NF-κB is located in the cytoplasm along with its inhibitor protein IκB. Inflammatory stimuli promote IκB degradation, which enables NF-κB translocation to the nucleus and binding to target gene promoters that regulate transcription of the pro-inflammatory cytokines. NF-κB is highly activated at sites of inflammation in multiple human cancers. There is accumulating evidence that NF-κB activation is correlated with prostate cancer development, prognosis, and castrate-resistant progression [9–12]. Cytokine IL-6 stimulates prostate cancer growth by stimulating the androgen receptor, and is involved in the development of bone metastasis [13]. Increased levels of NF-κB and IL-6 are implicated in the development of prostate cancer cell chemoresistance [14]. These combined studies suggest that NF-κB may be a promising target for improving chemotherapeutic efficacy in prostate cancer.

TBL1 and TBLR1, which are components of the nuclear hormone receptor corepressor (NCoR)/silencing mediator for retinoid and thyroid hormone receptors (SMRT) corepressor complexes, contain a Lis1 homology domain (LisH)/WD-40 motif, and have approximately 86% identity [15, 16]. Recent studies report that TBL1 and TBLR1 stabilize the quaternary structure of the NCoR/SMRT corepressor complex and bind to the tails of hypoacetylated histones H2B and H4, which target NCoR/SMRT complexes to chromatin [17]. The LisH domains of both TBL1 and TBLR1 are reported to be required for oligomerization, and contribute to the stable binding of NCoR/SMRT corepressor complexes to chromatin to completely repress target gene expression [18]. These results suggest that TBL1 and TBLR1 have important role in class I HDAC corepressor complex-mediated transcriptional repression. However, TBL1 and TBLR1 also were found to function as E3 ubiquitin ligase adaptors. In this capacity, they recruit the ubiquitin-conjugating/19S proteasome complex to the corepressor complex, which leads to corepressor degradation, and mediates the exchange of corepressors for coactivators in response to ligand binding/stimulation. These actions lead to specific nuclear receptor-mediated gene activation events [19, 20]. TBL1, TBLR1, and β-catenin recruit one another to Wnt target gene promoters to activate transcription in response to canonical Wnt signaling, and reversible SUMOylation of TBL1 and TBLR1 regulates β-catenin-mediated Wnt signaling [21, 22]. TBL1 is required for p65 recruitment to NF-κB target gene promoters in response to tumor necrosis factor alpha (TNF-α) [23]. These results suggest that TBL1 and TBLR1 function as transcriptional coactivators, and may have a role in the transcriptional activation of NF-κB target genes. However, the detailed mechanism of NF-κB-mediated transcriptional activation by TBL1 and TBLR1 corepressors was unknown.

In this study, we found that constitutive cytokine elevation in the androgen-independent prostate cancer (AIPC) cell lines PC-3 and C4-2B correlates with the SUMOylation levels of TBL1 and TBLR1. TBL1 and TBLR1 are SUMOylated *in vivo* in response to inflammatory stimuli. TBL1 and TBLR1 SUMOylation dissociate TBL1 and TBLR1 from the NCoR/HDAC3 corepressor complexes and induces formation of the TBL1^SUMO^-TBLR1^SUMO^-NF-κB complex, which ultimately leads to transcriptional activation of NF-κB target genes. Therefore, this study suggests a regulatory mechanism for elevated NF-κB-mediated inflammatory signaling in AIPCs via reversible SUMOylation of TBL1 and TBLR1.

## RESULTS

### TBL1 and TBLR1 SUMOylation and inflammatory cytokines are elevated in AIPC cells

**Figure 1:**
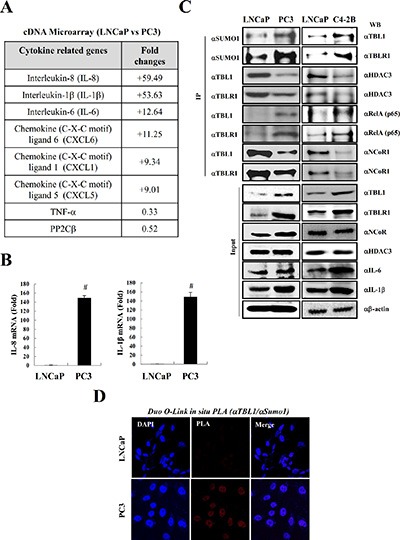
SUMOylation of TBL1 and TBLR1 is strongly elevated in androgen-independent prostate cancer cells enriched with inflammatory cytokines (**A**) Inflammatory cytokine levels are higher in PC-3 cells than in LNCaP cells. Changes in mRNA expression were evaluated by cDNA microarray analysis using the Illumina HumanRef-8 v3 Expression BeadChip. (**B**) Validation of cDNA microarray analysis by quantitative real-time PCR. Expression levels of each gene were analyzed by quantitative RT-PCR. Statistical significance was determined using Student's *t*-test; ^#^*P* < 0.01 *versus* LNCaP cell lines. (**C**) SUMOylation of TBL1 and TBLR1 levels are higher in PC-3 and C4-2B cells than in LNCaP cells. Immunoprecipitation analysis was performed using cell lysates, and immunoblotting was performed using the indicated antibodies. (**D**) Validation of TBL1 SUMOylation *in vivo* in LNCaP and PC-3 cells. Duo-link *in situ* PLA analysis was performed as described in Materials and methods with the indicated antibodies.

NF-κB is constitutively activated in prostate tumors and cell lines [5]. Therefore, we first examined the inflammatory cytokine levels in prostate cancer cell lines by performing cDNA microarrays using the androgen-dependent prostate cancer (ADPC) cell line LNCaP and the AIPC cell line PC-3. In agreement with a previous report [24], we observed that the pro-inflammatory cytokines IL-8, IL-1β, and IL-6 were strongly elevated in PC-3 cells compared with LNCaP cells (Figure 1A). Quantitative RT-PCR analysis verified the elevated cytokine levels in PC-3 cells (Figure 1B).

A recent study reported that the TBL1 corepressor acts as a cofactor for recruiting p65 to NF-κB target gene promoters, which eventually leads to the transcriptional activation of inflammatory cytokines [23]. Therefore, we explored the possibility that TBL1 and TBLR1 are involved in cytokine elevation in AIPC cells. First, we assessed TBL1 and TBLR1 levels in prostate cancer cells by performing western blot analysis. Immunoprecipitation analysis revealed that the interaction between TBL1/TBLR1 and RelA in PC-3 cells was strongly increased compared with that in LNCaP cells, and the TBL1 and TBLR1 protein levels in PC-3 cells also were higher than those in LNCaP cells (Figure 1C, left panel). TBL1 and TBLR1 SUMOylation caused TBL1 and TBLR1 dissociation from the NCoR corepressor complex [21]. Therefore, we next examined the relative association of TBL1 and TBLR1 with NCoR/HDAC3 corepressor complexes in PC-3 and LNCaP cells. TBL1 and TBLR1 association with NCoR/HDAC3 corepressor complexes were significantly lower in PC-3 cells than in LNCaP cells (Figure 1C, left panel). To verify these results, we performed Duo-link *in situ* proximity ligation assay (PLA) analysis, which enables the detection of protein interactions and modifications, and verified elevated SUMOylation levels of endogenous TBL1 in PC-3 cells (Figure 1D).

Due to their high metastatic potential resulting from their androgen-insensitive state, PC-3 cells have been less extensively studied than LNCaP cells for investigating biochemical changes in advanced prostate cancer. PC-3 cell line was established from bone metastasis of prostate cancer. Therefore, we selected the bone metastasis subline C4-2B, which was generated from parental LNCaP cells, to confirm whether these similar cell lines share the same biochemical features as PC-3 cells. The results showed that inflammatory cytokine levels were highly elevated in C4-2B cells compared with those in LNCaP cells, which was similar to the observed cytokine levels in PC-3 cells. The levels of TBL1 and TBLR1 SUMOylation and association of TBL1 and TBLR1 with RelA were higher in C4-2B cells than in PC-3 cells (Figure 1C, right panel). Collectively, these results suggest that constitutive activation of inflammatory signaling in AIPC cells correlates with TBL1 and TBLR1 SUMOylation.

### Inflammatory stimulation promotes TBL1 and TBLR1 SUMOylation

**Figure 2:**
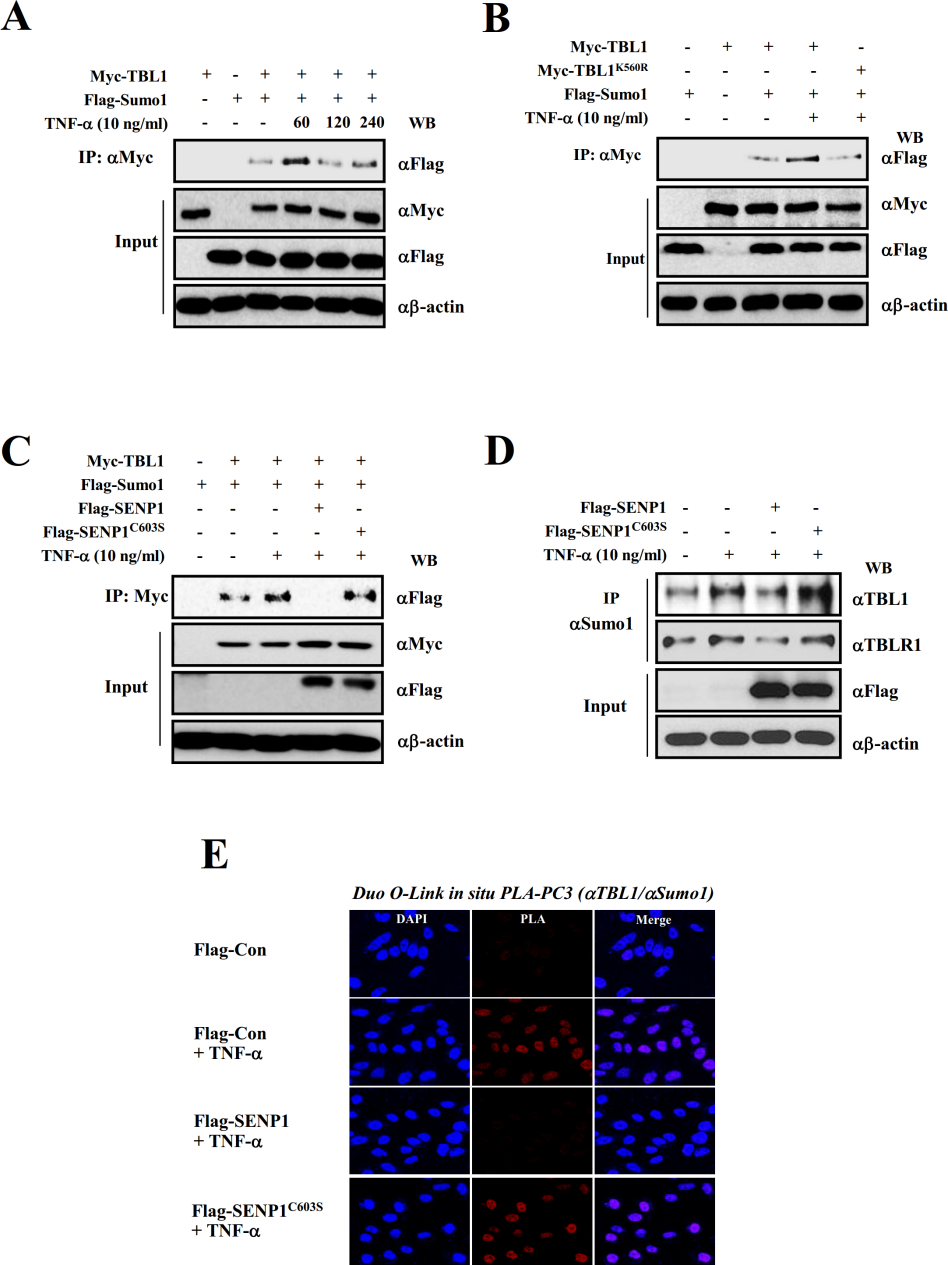
SENP1 suppresses TNF-α-induced TBL1 SUMOylation (**A**) TNF-α treatment induces TBL1 SUMOylation. PC-3 cells were co-transfected with indicated plasmids. Cells were treated with TNF-α for indicating time point and then harvested. Cell lysates were immunoprecipitated and subsequently immunoblotted with the indicated antibodies. (**B**) TNF-α induces SUMOylation of TBL1 at Lys560. Cells were co-transfected for 48 hours, treated with TNF-α for 1 hour and then harvested. Cell lysates were immunoprecipitated and subsequently immunoblotted with the indicated antibodies. (**C**) SENP1 deSUMOylates TBL1. (**D**) SENP1 suppresses TNF-α-induced SUMOylation of endogenous TBL1 and TBLR1. Cell lysates were immunoprecipitated and subsequently immunoblotted with the indicated antibodies. (**E**) Validation of SENP1-mediated TBL1 deSUMOylation *in vivo* in PC-3 cells. Permeabilized PC-3 cells were treated with TNF-α (10 ng/ml) for 1 hour and incubated with anti-TBL1 antibody, anti-SUMO antibody, and PLA probes (PLUS and MINUS).

Recent work showed that SUMO modification acts as a molecular switch that regulates corepressive and coactive functions of TBL1 and TBLR1 during Wnt signaling activation [21]. Therefore, we examined whether TBL1 and TBLR1 SUMOylation increases in response to inflammatory activation. Myc-TBL1 or Myc-TBLR1 was co-transfected with Flag-SUMO1 into PC-3 cells, the cells were treated with TNF-α, and immunoprecipitation assays were performed. In response to treatment, SUMOylation of both TBL1 and TBLR1 increased in response to TNF-α treatment (Figure 2A and Figure S1A). Our previous work showed that TBL1 and TBLR1 were SUMOylated on lysine 560 and lysine 497, respectively, during activation of Wnt signaling [21]. Therefore, we examined whether the TNF-α-mediated TBL1 SUMOylation site was identical with the Wnt signaling-induced SUMOylation site. The results indicated that TBL1^K560R^ and TBLR1^K497R^ mutants failed to form complexes with SUMO1, whereas wild-type TBL1 and TBLR1 did form SUMO1 complexes (Figure 2B and Figure S1B). These data confirm that TBL1 and TBLR1 are SUMOylated at Lys560 and Lys497, respectively, in response to inflammatory signaling.

The SUMO-processing enzyme SENP1 mediates deSUMOylation of TBL1 and TBLR1 [21]. We examined whether SENP1 deSUMOylates TBL1 and TBLR1. SENP1 efficiently removed SUMO from TBL1 and TBLR1, whereas inactive SENP1^C603S^ had negligible deSUMOylation activity on TBL1 and TBLR1 (Figure 2C and Figure S1C, lane 4 *versus* lane 5). We observed the same effects of SENP1 in deSUMOylation of endogenous TBL1 and TBLR1 (Figure 2D).

To corroborate inflammatory signaling-dependent TBL1 SUMOylation *in vivo*, we performed *in situ* PLA analysis by transfecting Flag-tagged SENP1^WT^ (wild-type) or SENP1^C603S^ (inactive mutant) into PC-3 cells. Consistently, endogenous TBL1 in PC-3 cells was efficiently SUMOylated in response to TNF-α. Ectopic expression of SENP1^WT^ efficiently reversed TNF-α-induced TBL1 SUMOylation, whereas SENP1^C603S^ overexpression had a negligible effect on TNF-α-induced TBL1 SUMOylation (Figure 2E). These combined results indicate that TBL1 and TBLR1 are SUMOylated in response to inflammatory stimuli, and are deSUMOylated by SENP1.

### TBL1 SUMOylation enhances NF-κB-mediated transcription

**Figure 3:**
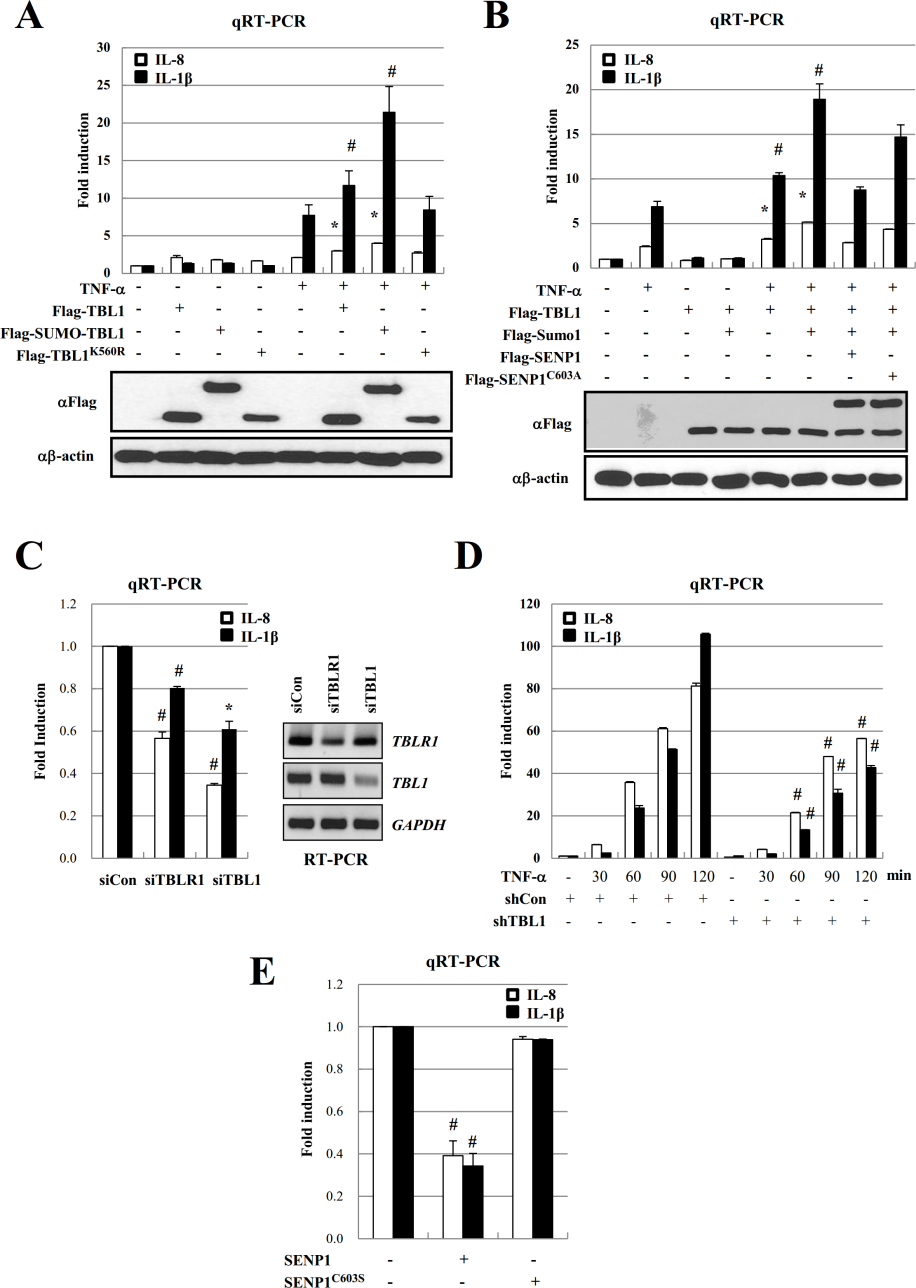
SENP1 inhibits TNF-α-induced NF-κB activation via deSUMOylation of TBL1 and TBLR1 (**A**) TBL1 SUMOylation potentiates its coactive function for NF-κB during inflammatory signaling. PC-3 cells were co-transfected with indicated plasmids for 48 hours and treated with 10 ng/ml TNF-α for 2 hours. Target gene mRNA levels were analyzed by qRT-PCR. Cell lysates were immunoblotted with the indicated antibodies. Statistical significance was determined using Student's *t*-test; *P < 0.05 and ^#^*P* < 0.01 *versus* treatment with TNF-α. (**B**) SENP1 reverses the effect of TBL1 SUMOylation on TNF-α-induced NF-κB activation. Co-transfected PC-3 cells were treated with TNF-α for 2 hours, mRNA levels were analyzed by qRT-PCR. Cell lysates were immunoblotted with the indicated antibodies. Statistical significance was determined using Student's *t*-test; *P < 0.05 and ^#^*P* < 0.01 *versus* treatment with TNF-α. (**C**) TBL1 or TBLR1 knockdown reduces cytokine levels in PC-3 cells. Cells were transfected with siRNA against TBL1 or TBLR1, and target gene mRNA levels were analyzed by qRT-PCR. Statistical significance was determined using Student's *t*-test; *P < 0.05 and ^#^*P* < 0.01 *versus* transfection with siControl. (**D**) TBL1 knockdown greatly reduces TNF-α-induced cytokine production in PC-3 cells. Lentiviral shTBL1-infected cells were treated with TNF-α, harvested, and mRNA levels were analyzed by qRT-PCR. Statistical significance was determined using Student's *t*-test; ^#^*P* < 0.01 *versus* infection with shControl samples. (**E**) SENP1 overexpression reduces cytokine levels in PC-3 cells. PC-3 cells were transfected with Flag-SENP1^WT^ or Flag-SENP1^C603S^, and mRNA levels of target genes were analyzed by qRT-PCR. Statistical significance was determined using Student's *t*-test; *P < 0.05 and ^#^*P* < 0.01 *versus* Control transfection (without SENP1^WT^ and SENP1^C603S^).

Inflammatory cytokines and SUMOylated TBL1/TBLR1 are highly elevated in PC-3 cells. Therefore, we examined the effect of TBL1 SUMOylation on NF-κB-mediated transcription of cytokine genes. Ectopic expression of SUMO-conjugated TBL1 enhanced TNF-α-induced *IL-8* and *IL-1β* gene transcription compared to that of unconjugated TBL1 (Figure 3A). Expression of SENP1^WT^ efficiently reversed the effect of Sumo-conjugated TBL1, whereas expression of inactive SENP1^C603S^ failed to reverse the effect (Figure 3B). These combined results indicate a crucial role for TBL1 SUMOylation in NF-κB-mediated activation of cytokine gene transcription.

Next, we tested whether TBL1 or TBLR1 knockdown suppresses transcriptional activation of cytokine gene expression in PC-3 cells. Treatment of PC-3 cells with either siTBL1 or siTBLR1 substantially reduced the basal levels of *IL-8* and *IL-1β* (Figure 3C). Treatment of PC-3 cells with TNF-α time-dependently increased *IL-8* and *IL-1β* gene expression; however, TBL1 knockdown greatly reduced TNF-α-dependent cytokine gene expression in PC-3 cells (Figure 3D). Consistently, overexpression of SENP1^WT^, but not SENP1^C603S^, efficiently suppressed the cytokine transcript levels in PC-3 cells (Figure 3E), indicating that TBL1 and TBLR1 SUMOylation constitutively enhance inflammatory cytokine production in PC-3 cells.

### Inflammatory stimulation induces formation of the TBL1^SUMO^-TBLR1^SUMO^-NF-κB activator complex

**Figure 4:**
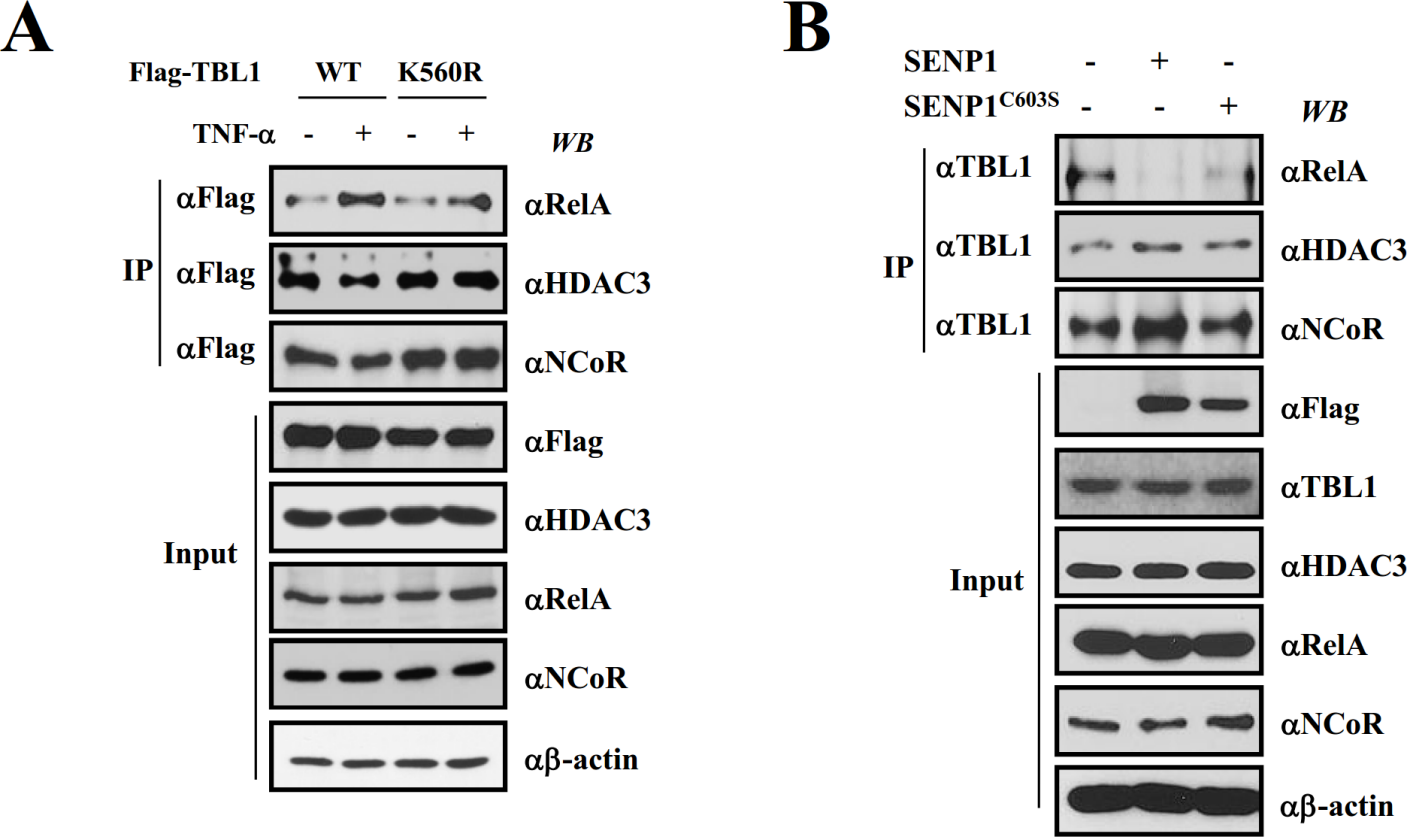
TBL1 SUMOylation enhances formation of the TBL1^SUMO^-NF-κB complex (**A**) TNF-α-induced TBL1 SUMOylation at Lys560, is required for enhanced TBL1 association with NF-κB. PC-3 cells were co-transfected with the indicated plasmids, treated with or without TNF-α for 1 hour, and then harvested. Cell lysates were immunoprecipitated and subsequently immunoblotted with the indicated antibodies. (**B**) SENP1-mediated TBL1 deSUMOylation induces disassembly of the TBL1-NF-κB complex. PC-3 cells were co-transfected with the indicated plasmids. Cell lysates were immunoprecipitated and subsequently immunoblotted with the indicated antibodies.

SUMOylation induces TBL1 and TBLR1 dissociation from the NCoR corepressor complex, which corresponds with formation of the TBL1-TBLR1-β-catenin coactivator complex [21]. Therefore, we examined whether SUMOylation increases formation of the TBL1-TBLR1-NF-κB complex in response to TNF-α treatment. TNF-α treatment increased the association of wild-type TBL1 with NF-kB (greek letter), whereas TNF-α reduced the interaction between TBL1 and HDAC3 or NCoR (Figure 4A). Figure S2 showed same effect of TNF-α on TBLR1 with NF-κB, HDAC3 or NCoR association. These results verify that TBL1 and TBLR1 assembly with NF-κB depends on TBL1 SUMOylation. Mutation of TBL1 SUMOylation sites attenuated TNF-α-induced TBL1 association with RelA, and promoted the strong interaction of mutant TBL1 with NCoR or HDAC3. These results indicate that SUMOylation of TBL1 and TBLR1 at Lys560 and Lys497, respectively, is required for TNF-α-induced TBL1 and TBLR1 assembly with NF-κB and NCoR corepressor complex disassembly.

We tested the effect of SENP1 overexpression on formation of the TBL1-NF-κB complex. Active SENP1 substantially induced TBL1 dissociation from NF-κB and association with the NCoR/HDAC3 corepressor complex, whereas inactive SENP1^C603S^ did not have these effects (Figure 4B). These combined results indicate that TBL1/TBLR1 SUMOylation has a crucial role in formation of the TBL1^SUMO^-TBLR1^SUMO^-NF-κB coactivator complex during inflammatory stimulation.

### TBL1 and TBLR1 SUMOylation enhances recruitment of the TBL1^SUMO^-TBLR1^SUMO^-NF-κB complex to the *IL-6* and *IL-8* promoter

**Figure 5:**
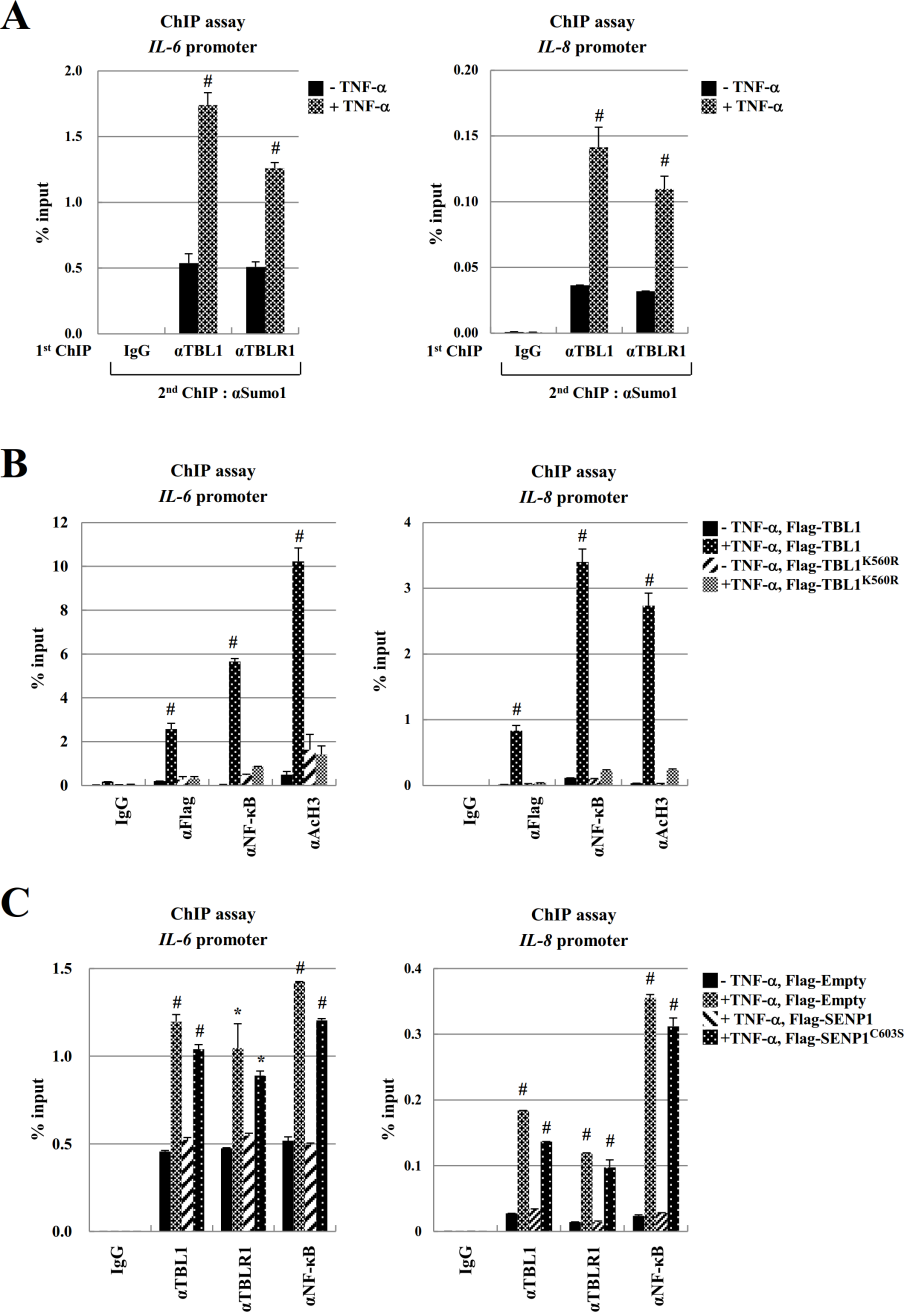
TBL1 and TBLR1 SUMOylation enhance recruitment of the TBL1-TBLR1-NFκB complex to the *IL-6* and *IL-8* promoter (**A**) TNF-α treatment induces recruitment of the TBL1^SUMO^-TBLR1^SUMO^-NF-κB complex to the *IL-6* and *IL-8* promoter regions. Cells were treated with 10 ng/ml TNF-α for 1 hour, ChIP and reChIP assays were performed with the indicated antibodies. Error bars represent standard deviation (SD, *n* = 3). Statistical significance was determined using Student's *t*-test; ^#^*P* < 0.01 *versus* treatment without TNF-α. (**B**) TBL1 SUMOylation enhances NF-κB binding to chromatin at the *IL-6* and *IL-8* promoter. PC-3 cells were transfected with the indicated plasmids, followed by ChIP assays with the indicated antibodies. Error bars represent SD (*n* = 3). Statistical significance was determined using Student's *t*-test; ^#^*P* < 0.01 *versus* Flag-TBL1 without TNF-α treatment. (**C**) SENP1-mediated TBL1 and TBLR1 deSUMOylation induces dissociation of the TBL1-TBLR1-NFκB complex from promoters of NF-κB target genes in PC-3 cells. PC-3 cells were transfected with plasmids carrying SENP1^WT^ and/or SENP1^C603S^. Cell lysates were immunoprecipitated and then used for performing ChIP assays with the indicated antibodies. Error bars represent SD (*n* = 3). Statistical significance was determined using Student's *t*-test; *P < 0.05 and ^#^*P* < 0.01 *versus* transfection of Flag-TBL1 without TNF-α treatment.

During immune responses, NF-κB is translocated into the nucleus where it binds to specific sites of cytokine genes such as *IL-6*, *IL-8*, and *IL-1β* [24–26]. Recent work also showed that TBL1 and NF-κB specifically bind to the promoter regions of NF-κB target genes in an inflammation signal-dependent manner. Therefore, we examined whether SUMOylation of TBL1 and TBLR1 enhances recruitment of the TBL1-TBLR1-NF-κB complex to the NF-κB-binding sites of cytokine genes. TNF-α treatment efficiently induced NF-κB and TBL1-TBLR1 binding to the NF-κB-binding region of *IL-6 and IL-8*. Conversely, reChIP experiments showed that TBL1^SUMO^-TBLR1^SUMO^ formed a complex with NF-κB, and the complex bound to the NF-κB-binding region of *IL-6 and IL-8* (Figure 5A). Overexpression of wild-type TBL1 enhanced NF- κB binding to the *IL-6* promoter and dramatically increased histone H3 acetylation in this region, whereas SUMOylation-defective TBL1 mutants did not exhibit these effects. We observed the same effects in *IL-8* promoter ChIP assay (Figure 5B). These data suggest that TBL1 SUMOylation triggers formation of an NF-κB-TBL1 complex in the promoter regions of NF-κB target genes. ChIP assays indicated that SENP1^WT^ overexpression induced TBL1-TBLR1-NF-κB complex dissociation from the NF-κB-binding sites of cytokine genes, whereas overexpression of inactive SENP1^C603S^ did not significantly affect TBL1-TBLR1-NF-κB complex binding to cytokine genes. These combined results indicate that TBL1 and TBLR1 deSUMOylation attenuate cytokine signaling by inducing TBL1-TBLR1-NF-κB complex dissociation from the NF-κB-binding sites of cytokine genes (Figure 5C).

### TBL1 and TBLR1 deSUMOylation suppress PC-3 proliferation

**Figure 6:**
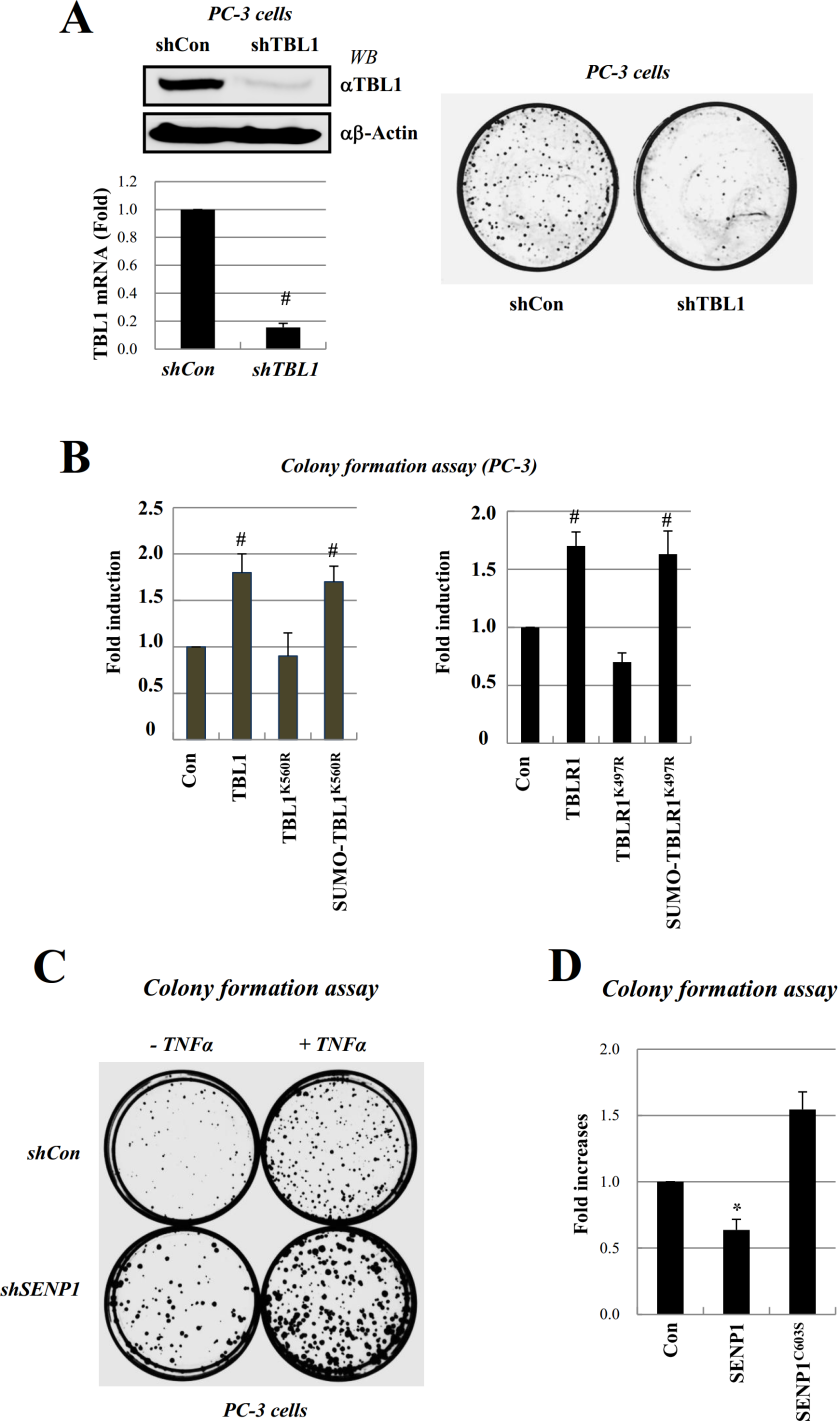
Inhibition of TBL1 and TBLR1 SUMOylation reduces PC-3 cell growth (**A**) TBL1 knockdown greatly reduces anchorage-independent PC-3 cell proliferation. PC-3 cells were infected with lentiviral shTBL1 to generate TBL1 knockdown lines. Knockdown efficiency was measured by RT-PCR and qRT-PCR (left panel) compared to shCon cell lines; shCon or shTBL1 cells were seeded with the same cell numbers, and plates were photographed after 3 weeks. (**B**) TBL1 and TBLR1 SUMOylation enhance tumorigenic growth of PC-3 prostate cancer cells. PC-3 cells were co-transfected with TBL1 (left panel) or TBLR1 (right panel) plasmids, and the numbers of cells in soft agar were determined under a microscope 2 days after transfection. Data represent the means ± SD of at least three independent experiments. Statistical significance was determined using Student's *t*-test; ^#^*P* < 0.01 *versus* Control. (**C**, **D**) SENP1 suppresses tumorigenic growth of PC-3 cells. SENP1 knockdown greatly increased anchorage-independent PC-3 cell proliferation (C). PC-3 cells were infected with lentiviral shSENP1 to generate SENP1 knockdown cell lines; shCon or shSENP1 cells were seeded with the same cell numbers onto media with or without TNF-α (changed every day), allowed to grow for 3 weeks, and then plates were photographed. PC-3 cells were transfected with SENP1^WT^ or SENP1C^603S^ as indicated, and the numbers of cells in soft agar were determined under a microscope. Data represent the means ± SD of at least three independent experiments. Statistical significance was determined using Student's *t*-test; *P < 0.05 versus Control (D).

PC-3 cell line is a representative model for androgen-independent prostate cancer. NF-κB is constitutively active in PC-3 and DU145 AIPC cell lines, whereas only very low levels of NF-κB activity are detected in the LNCaP ADPC cell line. These observations suggest that constitutive NF- kB activation may have a role in the survival of AIPC cells [10, 11]. We examined whether reduced TBL1 levels or TBL1-TBLR1 deSUMOylation attenuated anchorage-independent growth, which typically correlates with *in vivo* tumorigenic phenotype. PC-3 proliferation was substantially lower after TBL1 knockdown than in cells transfected with control short hairpin RNA (shRNA) (Figure 6A). Overexpression of TBL1^WT^ or TBLR1^WT^ enhanced PC-3 cell growth compared with overexpression of TBL1^K560R^ or TBLR1^K497R^ (Figure 6B, left panel). Expression of TBL1^K560R^ reduced PC-3 cell growth and colony formation, whereas expression of SUMO-conjugated TBL1^K560R^ restored PC-3 cell growth and colony formation (Figure 6B). TBLR1 behaved similarly to TBL1 in these assays (Figure 6B, right panel). Overexpression of wild-type SENP1^WT^, but not inactive SENP1^C603S^, greatly reduced anchorage-independent PC-3 cell growth, indicating the functional relevance of TBL1-TBLR1 SUMOylation for tumorigenic growth of PC-3 cells (Figure 6C, 6D). These combined results indicate that TBL1-TBLR1 SUMOylation enhances NF-κB-mediated inflammatory signaling and proliferation of androgen-independent prostate cancer cells.

## DISCUSSION

NF-κB, a transcriptional factor, is strongly activated at sites of inflammation, which plays critical role in variety of gene expression including cytokines [24]. A recent study reported that TBL1 was required for p65 recruitment to NF-κB-targeted gene promoters in response to TNF-α [23], which suggests an additional role for TBL1 as a transcriptional activator. In that study, TBL1 recruitment to NF-κB-targeted gene promoters was considered a critical step for the transcriptional activation of target genes. However, the molecular mechanism underlying NF-κB activation by the well-known TBL1 corepressor has not been fully elucidated.

In this study, we present evidence that SUMOylation of TBL1 and TBLR1 enhances association with NF-κB in response to TNF-α, and induce dissociation of the NCoR-TBL1-TBLR1 corepressor complex. Our group previously reported that SUMOylation of TBL1 and TBLR1 promotes β-catenin-mediated Wnt signaling via disassembly of NCoR-TBL1 corepressor complex. We observed that TBL1 and TBLR1 are SUMOylated at lysine 560 and lysine 497, respectively, in response to cytokine signaling, and are deSUMOylated by SENP1. SENP1 overexpression suppresses the coactive function of TBL1 and TBLR1 in NF-κB-mediated transcriptional regulation of cytokine gene expression, which is similar to the effects of Wnt signaling. Therefore, this study provides additional evidence that TBL1 and TBLR1 SUMOylation induce the functional exchange of TBL1 and TBLR1 from corepressor to coactivator activity via disassembly of the NCoR corepressor complex.

Chronic inflammation is linked to carcinogenesis in several organ systems [1], and prostate cancer is strongly correlated with chronic inflammation [5]. There is accumulating evidence that elevated serum IL-6 levels are associated with advanced tumor stages in patients with prostate cancer [27]. Elevated NF-κB and IL-6 levels have been correlated with drug resistance of prostate cancer cells [9, 11]. For example, IL-6 is an autocrine and paracrine growth factor for prostate cancer cell lines and functions as a resistance factor for cisplatin-mediated cytotoxicity [14]. Clinical trials reported that antibody-targeted IL-6 therapy may be useful for improving the performance of other chemotherapy agents during treatment of metastatic castration-resistant prostate cancer [28]. These combined results suggest that suppression of IL-6 levels may be a potential therapeutic strategy for treating prostate cancer. We also observed constitutively elevated levels of cytokines IL-6, IL-8, and IL-1β in PC-3 compared with the levels in LNCaP cells, indicating that NF-κB is constitutively activated in PC-3 cells. This result is consistent with previous studies reporting that NF-κB is constitutively activated in androgen-independent PC-3 prostate cancer cells but not in androgen-dependent LNCaP prostate cancer cells. TBL1/TBLR1 SUMOylation is elevated in AIPC PC-3 cells compared with that in LNCaP cells and the LNCaP-derived AIPC cell line C4-2B. SUMOylation of TBL1 and TBLR1 is closely correlated with NF-κB activity. Furthermore, this study shows direct evidence that sumolyated TBL1 and TBLR1 are recruited to promoters of both IL-6 and IL-8 under inflammation, whereas defective form of SUMOylation of TBL1, TBL1K560R, fails to recruit NF-kB to the promoters, which means SUMOylated TBL1 and TBLR1 upregulate the IL-6 and IL-8 promoter through recruiting NF-kB to the kB binding regions of two genes.

Overexpression of active SENP1, but not inactive SENP1, dramatically suppresses TBL1/TBLR1-mediated NF-κB activity and PC-3 cell proliferation. These results suggest another possible link between TBL1/TBLR1 SUMOylation and inflammatory signaling during tumorigenic growth of AIPC cells. There is additional evidence that the E2 ligase Ubc9 is strongly expressed in primary prostate tumor and cancer cells but not in metastatic prostate tumors [29, 30]. Our results indicate that SUMOylation of TBL1 and TBLR1 may contribute to AIPC cell proliferation after androgen withdrawal. Further studies are needed to completely elucidate these processes.

In conclusion, our study identified a SUMOylation-dependent regulatory mechanism of inflammatory signaling in androgen-independent prostate cancer cells. TBL1 and TBLR1 were reversibly SUMOylated in response to inflammatory stimuli, which induced transcriptional activation of inflammatory cytokines by formation of the TBL1^SUMO^-TBLR1^SUMO^-NF-κB complex. We showed that inhibition of TBL1-TBLR1 SUMOylation significantly suppressed cytokine production and proliferation of androgen-independent prostate cancer cells. Therefore, our study provided important insights into inflammation-promoted prostate tumorigenesis and targets for treating AIPC.

## MATERIALS AND METHODS

### Cell culture, plasmids, and reagents

C4-2B cell lines were purchased from Leland Chung's group at MD Anderson (Houston, TX, USA). C4- 2B, LNCaP, and PC-3 human prostate cancer cell lines were cultured in Roswell Park Memorial Institute 1640 medium (RPMI-1640, Hyclone Cell Culture Media, GE Healthcare, UT, USA) supplemented with 10% (v/v) fetal bovine serum (FBS, Hyclone Cell Culture Media, GE Healthcare), and 1% antibiotics and antimycotics (Hyclone Cell Culture Media, GE Healthcare). All cell lines were cultured at 37°C in 5% CO_2_. Human recombinant TNF-α was purchased from ProSpec (Rehovot, Israel), and prepared as 100 μg/ml stocks in sterile H2O (18 MΩ·cm). 10 ng/ml concentration of TNF-α was used to treat the cells during indicated time point. Preparation of full-length TBL1, TBL1^K560R^, SUMO-TBL1^K560R^, TBLR1, TBLR1^K497R^, SUMO-TBLR1^K497R^, and SUMO1 was described previously [21]. SENP1 clones were obtained from the 21 C Human Frontier Human Gene Bank (Daejeon, Korea), and were subcloned by inserting the PCR amplification product into the plasmid pSG5-KF2M1. All plasmid constructs were verified by DNA sequencing. The retroviral-based mammalian expression vector pBabe was used to stably express wild-type TBL1/TBLR1, mutant TBL1/TBLR1, SUMO-fused TBL1/TBLR1, SENP1, and SENP1^C603S^ protein in PC-3 cells. PC-3 cells stably expressing either SENP1 or SENP1^C603S^ were generated by puromycin selection.

### Lentiviral short hairpin RNAs

We used shRNAs to establish stable PC-3 cells with reduced TBL1 or SENP1 expression. First, two pairs of commercially available oligonucleotides encoding each target-specific shRNA were purchased (MISSION shRNA, Sigma-Aldrich). Next, we prepared lentiviral particles using pLKO.1-PURO targeted shRNA, using three plasmid co-transfections according to the manufacturer's instructions (Invitrogen, Carlsbad, CA, USA). Then, PC-3 cells were transfected with lentivirus, incubated for 3 days, and lentivirus was isolated from culture medium and concentrated with a Centricon-Plus-20 column (Millipore, Billerica, MA, USA). Lentivirus PURO shRNA was generated as a control.

### Immunoprecipitation and immunoblotting

Cells were plated at 60–70% confluency and transfected using Lipofectamine 2000 (Invitrogen). Cells were lysed in lysis buffer (50 mM Tris-HCl, pH 7.5, 0.3 M NaCl, 1% NP-40, 1 mM PMSF, and protease inhibitor mixture). Lysates were clarified by centrifugation at 13,000 rpm for 20 min at 4°C. Protein concentrations were determined spectrophotometrically using the 660 nm protein assay reagent (Pierce, Rockford, USA). Immunoprecipitation (IP) analysis was performed using 500 μg of whole cell lysate, 15 μl of protein plus A/G agarose (Santa Cruz Biotechnology), Flag-M2 agarose (Sigma), and commercial antibodies. IP assays were incubated overnight with gentle rotation at 4°C. Then, immunoprecipitates were washed 3 times in washing buffer for 5 min. After the last wash, immunoprecipitates were boiled in 5 × SDS loading buffer. Immunoprecipitated proteins were analyzed by western blotting. All antibodies were commercially available except for antibodies against TBL1, TBLR1, and NCoR1. Commercially available antibodies were used at dilutions of 1:500 to 1:1,000 [15, 16]. Monoclonal anti-HDAC3 was obtained from BD Transduction Laboratories (Lexington, KY, USA) and was used at a dilution of 1:3,000. Polyclonal anti-HDAC3, monoclonal anti-Flag, and anti-β-actin antibodies were obtained from Sigma and used at a dilution of 1:10,000. Anti-SUMO1 and polyclonal anti-Myc antibodies were obtained from Santa Cruz Biotechnology (Santa Cruz, CA, USA) and used at a dilution of 1:500 to 1:2,000. Positive signals were developed using Lumi-Light Western Blotting Substrate (Roche, Indianapolis, IN, USA) according to the manufacturer's instructions.

### Real-time RT-PCR

Total RNA was isolated from LNCaP, PC-3, and C4- 2B cell lines using TRIzol reagent (Takara, Japan). Then, cDNAs were synthesized from 3 μg of total RNA using Oligo-dT and M-RT reverse transcriptase (Chromogen, Seoul, Korea). Real-time qRT-PCR assays were performed using ABI PRISM 7000 Sequence Detection System instrumentation and software (Applied Biosystems, Foster City, CA) according to the manufacturer's instructions with minor modifications. Briefly, the appropriate amount of reverse transcription reaction mixture was amplified with specific primers using SYBR green PCR master mix (Applied Biosystems). Target gene expression levels were determined by generating a five-point serial standard curve. The singularity and specificity of amplifications were checked using dissociation analysis software. All reactions were performed in triplicate. Concentrations of the RNA samples were normalized by determining the GAPDH mRNA level. The following primers were used: for IL- 6, 5′-CCCCCAGGAGAAGATTCCAA-3′ (forward) and 5′-GCT GCTTTCACACATGTTACTCTTG-3′ (reverse); for IL-1β, 5′-ACCTGAGCTCGCCAGTGAA-3′ (forward) and 5′-TCG GAGATCGTAGCTGGAT-3′ (reverse); for IL-8, 5′-TCCTTG TTCCACTGTGCCTTG-3′ (forward) and 5′-TGCTTCCAC ATGTCCTCACAAC-3′ (reverse); for TBL1, 5′-CATCTCCA TTCTCCAGAAGGG-3′ (forward) and 5′-GTGACAGGG ACTCTATGG-3′ (reverse); for GAPDH, 5′-GATGGCATGG ACTGTGGTCA-3′ (forward) and 5′- GCAATGCCTCCTG CACCACC -3′ (reverse).

### Chromatin immunoprecipitation assay

Chromatin immunoprecipitation (ChIP) assays were performed with the indicated antibodies as described previously [18], but without SDS in all buffers. Eluted DNA was amplified with specific primers using SYBR green PCR master mix (Applied Biosystems). The primers used for the *IL-6* gene promoter NF- κB binding site were 5′-CCCACCCTCCAACAAAGATT-3′ (forward) and 5′-GCTCCAGAGCAGAATGAGCTA-3′ (reverse); for *IL-8* gene promoter were 5′-GGGCCATC AGTTGCAAATC-3′ (forward) and 5′-GGAAGAAACCA CCGGAAGGAA-3′ (reverse).

### Colony formation

Cells were suspended in RPMI-1640 medium containing 10% FBS and 0.3% SeaKem low melting temperature agarose. A total of 1 × 10^3^ cells in a volume of 15 ml were plated in six-well plates over a 1.5-ml layer of solidified RPMI-1640 containing 10% FBS and 0.6% agarose. The plates were incubated at 37°C for 3 weeks, and then the colonies formed on each plate were photographed from three different views. The numbers and sizes of colonies on each plate were counted and measured.

### Duo-link *in situ* proximity ligation assay analysis

Duo-link *in situ* proximity ligation assay (PLA) analysis was performed according to the manufacturer's instructions (Olink Biosciences, Uppsala, Sweden). Briefly, paraformaldehyde-fixed cells were washed with PBS, incubated in 1.5% hydrogen peroxide for 15 min, washed, and blocked with blocking solution. Primary rabbit antibody was applied, cells were incubated with PLUS and MINUS secondary PLA probes against rabbit IgG only, or against both rabbit and mouse IgG, and then samples were subjected to hybridization, ligation, and amplification. Cells were mounted with Duo-link mounting medium and examined using an Olympus FluoView FV1000 Confocal Microscope (Olympus Corp., Tokyo, Japan).

### Statistical analysis

Statistical analyses were performed using Student's *t*-test with Bonferroni for multiple comparisons. A *p*-value less than 0.05 was considered as statistically significant.

## SUPPLEMENTARY MATERIALS TABLES



## Abbreviations

TBL1: transducing β-like 1X-linked protein
TBLR1: Transducin β-like 1X-linked receptor 1
NF-κB: nuclear factor kappa-light-chain-enhancer of activated B cells
SENP1: Sentrin-specific protease 1
HDAC: Histone Deacetylase.
