# Supplementary material for: SUMOylation of TBL1 and TBLR1 promotes androgen-independent prostate cancer cell growth
Source: Oncotarget. 2016 Apr 26;7(27):41110–22. doi: 10.18632/oncotarget.9002 (PMC5173046; doi:10.18632/oncotarget.9002)
Supplement: Supplementary file 1 [file oncotarget-07-41110-s001.pdf]

SUMOylation of TBL1 and TBLR1 promotes androgen-independent prostate cancer cell growth

Supplementary Materials

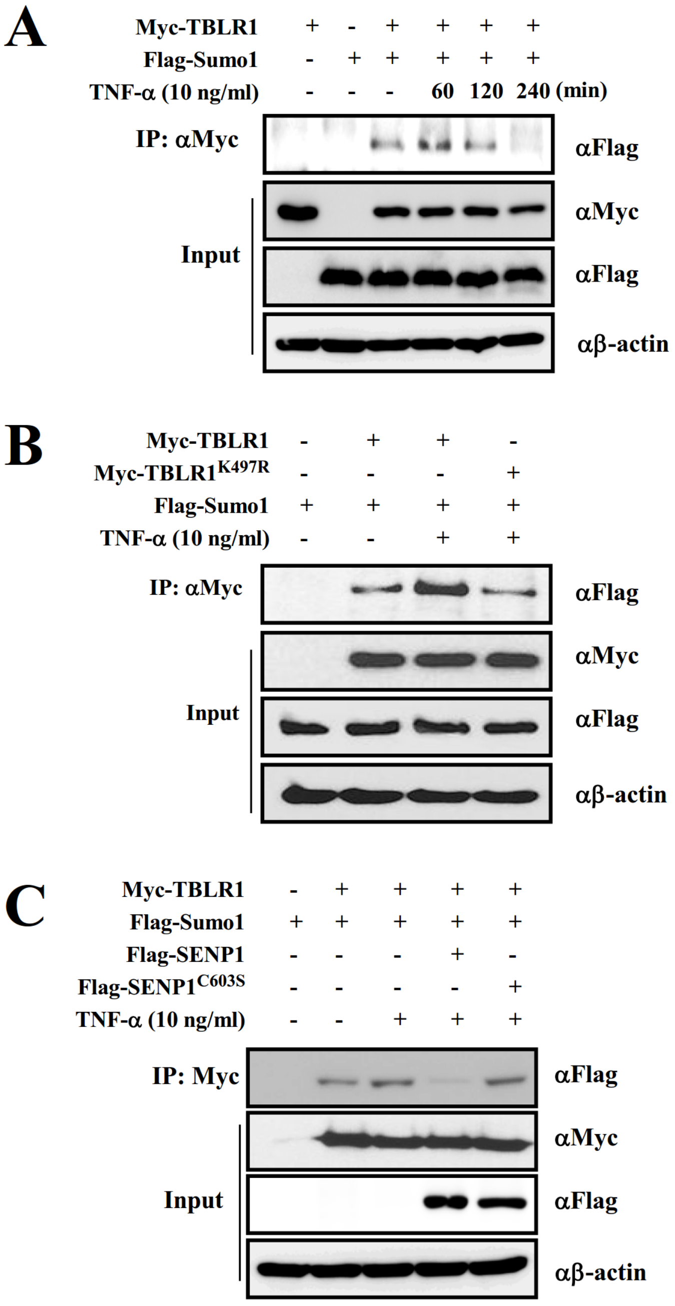

Supplementary Figure S1: SENP1 suppresses TNF-α-induced TBLR1 SUMOylation. (**A**) TNF-α treatment induces TBLR1 SUMOylation. Cells were treated with TNF-α for indicating time point and then harvested. Cell lysates were immunoprecipitated and subsequently immunoblotted with the indicated antibodies. (**B**) TNF-α induces SUMOylation of TBLR1 at Lys497. Cells were co-transfected for 48 hours, treated with TNF-α for 1 hour and then harvested. Cell lysates were immunoprecipitated and subsequently immunoblotted with the indicated antibodies. (**C**) SENP1 deSUMOylates TBLR1.

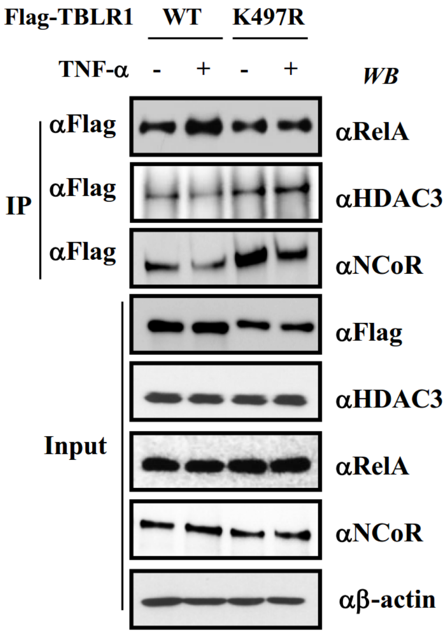

Supplementary Figure S2: TBLR1 SUMOylation enhances formation of the TBLR1^{SUMO}-NF-κB complex. TNF-α-induced TBLR1 SUMOylation at Lys497 is required for TBLR1-NF-κB interaction. PC-3 cells were co-transfected with the indicated plasmids, treated with or without TNF-α for 1 hour, and then harvested. Cell lysates were immunoprecipitated and subsequently immunoblotted with the indicated antibodies.
